# Machine Learning Representation of Loss of Eye Regularity in a *Drosophila* Neurodegenerative Model

**DOI:** 10.3389/fnins.2020.00516

**Published:** 2020-06-04

**Authors:** Sergio Diez-Hermano, Maria D. Ganfornina, Esteban Vegas-Lozano, Diego Sanchez

**Affiliations:** ^1^Instituto de Biologia y Genetica Molecular-Departamento de Bioquimica y Biologia Molecular y Fisiologia, Universidad de Valladolid-CSIC, Valladolid, Spain; ^2^Departamento de Biodiversidad, Ecologia y Evolucion, Unidad de Biomatematicas, Universidad Complutense, Madrid, Spain; ^3^Departamento de Genetica, Microbiologia y Estadistica, Universidad de Barcelona, Barcelona, Spain

**Keywords:** *Drosophila melanogaster*, neurodegeneration, rough eye phenotype, spinocerebellar ataxia, machine learning, classification, deep learning

## Abstract

The fruit fly compound eye is a premier experimental system for modeling human neurodegenerative diseases. The disruption of the retinal geometry has been historically assessed using time-consuming and poorly reliable techniques such as histology or pseudopupil manual counting. Recent semiautomated quantification approaches rely either on manual region-of-interest delimitation or engineered features to estimate the extent of degeneration. This work presents a fully automated classification pipeline of bright-field images based on orientated gradient descriptors and machine learning techniques. An initial region-of-interest extraction is performed, applying morphological kernels and Euclidean distance-to-centroid thresholding. Image classification algorithms are trained on these regions (support vector machine, decision trees, random forest, and convolutional neural network), and their performance is evaluated on independent, unseen datasets. The combinations of oriented gradient + gaussian kernel Support Vector Machine [0.97 accuracy and 0.98 area under the curve (AUC)] and fine-tuned pre-trained convolutional neural network (0.98 accuracy and 0.99 AUC) yielded the best results overall. The proposed method provides a robust quantification framework that can be generalized to address the loss of regularity in biological patterns similar to the *Drosophila* eye surface and speeds up the processing of large sample batches.

## Introduction

*Drosophila melanogaster* stands out as one of the key animal models in today’s modern genetic studies, with an estimated 75% of human disease genes having orthologs in flies (Reiter et al., 2001). Its growth as a powerful experimental model of choice has been supported by the wide array of genetic and molecular biology tools designed with the fruit fly in mind (Johnston, 2002), easing the creation of genetic deletions, insertions, knock-downs, and transgenic lines. Fly biologists have greatly contributed to our knowledge of mammalian biology, making *Drosophila* the historical premier research system in the fields of epigenetics, cancer molecular networks, neurobiology, and immunology (Wangler et al., 2015). The relative simplicity of *Drosophila* genetics (four pairs of homologous chromosomes, in contrast to 23 in humans) and organization (i.e., ∼2 × 10^5^ neurons in opposition to roughly 10^11^ neurons in humans) makes the fruit fly an especially well-suited model for the analysis of subsets of phenotypes associated with complex disorders.

Specifically, the retinal system in *Drosophila* has been widely used as an experimental setting for high-throughput genetic screening and for testing molecular interactions (Thomas and Wassarman, 1999). Eye development is a milestone in the *Drosophila* life cycle, with a massive two-thirds of the essential genes in the fly genome required at some point during the process (Thaker and Kankel, 1992; Treisman, 2013). Therefore, it constitutes an excellent playground to study the genetics underlying general biological phenomena, from the basic cellular and molecular functions to the pathogenic mechanisms involved in multifactorial human diseases, such as diabetes or neurodegeneration (Garcia-Lopez et al., 2011; Lenz et al., 2013; He et al., 2014).

**FIGURE 1:**
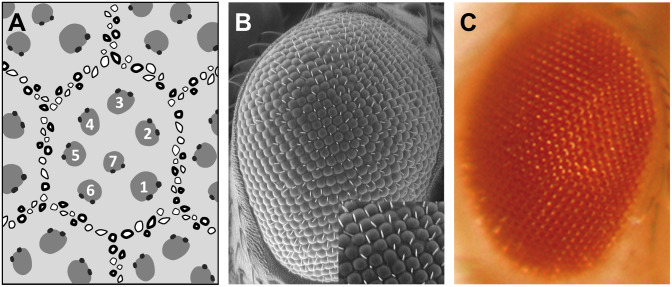
*Drosophila* compound eye structure. Different eye imaging techniques demonstrating the hexagonal packing of the ommatidia and the trapezoidal arrangement of the photoreceptors. **(A)** Schematic representation of a tangential section through the eye. Numbers depict photoreceptors. **(B)** Scanning electron micrograph (SEM). Higher magnification view in inset. **(C)** Bright field microscope picture.

The fruit fly compound eye is a biological system structured as a stereotypic array of 800 simple units, called ommatidia, which display a highly regular hexagonal pattern (Figure 1). This strict organization precisely allows to evaluate the impact of altered gene expression and mutated proteins on the external eye morphology and to detect subtle alterations on the ommatidia geometry due to cell degeneration. One special type of cellular deterioration largely studied using *Drosophila* retina encompasses polyglutamine-based neurodegenerative diseases, namely, Huntington’s and spinocerebellar ataxias (SCA) (Ambegaokar et al., 2010).

**FIGURE 2:**
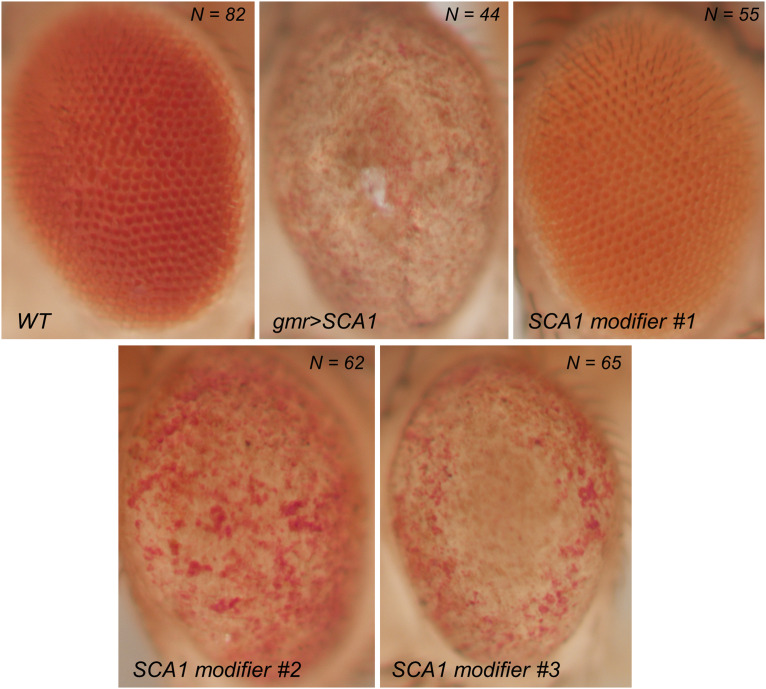
Bright field photographs of rough eye phenotype surfaces. SCA1 gene modifiers can be tested on the fly eye using the UAS/Gal4 system. Complete loss of surface regularity and depigmentation can be appreciated between the WT and SCA1 phenotypes. SCA1 modifiers show intermediate levels of degeneration.

The overexpression of polyQ-expanded proteins *via* the UAS/Gal4 system in the fly retina results in a depigmented, rough eye phenotype caused by the loss of interommatidial bristles (see the wild-type pattern in the inset of Figure 1B), ommatidial fusion, and necrotic tissue (Figure 2). The vast majority of studies assessing the rough eye morphology rely on qualitative examination (i.e., visual inspection) of its external appearance to manually rank and categorize mutations based on their severity (Roederer et al., 2005; Bilen and Bonini, 2007; Cukier et al., 2008). Even though evident degenerated phenotypes are easily recognizable, weak modifiers or subtle alterations may go undetected for the naked eye. Quantitative approaches addressing this issue involve histological preparations from which to evaluate the retinal thickness and the regularity of the hexagonal array or scoring scales for the presence of expected features in the retinal surface (Jonshon and Cagan, 2009; Jenny, 2011; Caudron et al., 2013; Mishra and Knust, 2013; Song et al., 2013). Recently, there have been efforts to fully computerize the analysis of *Drosophila*’s rough eye phenotype in bright-field and scanning electron micrograph (SEM) images in the form of ImageJ plugins, called FLEYE and Flynotiper (Diez-Hermano et al., 2015; Iyer et al., 2016). Whereas both methods propose automatized workflows, the former prompts the user to manually delimit the region of interest (ROI) to extract the hand-crafted features from it, which serve as input to a statistical model and finally output a regularity index (IREG) to the user. The second method relies upon a single engineered feature and lacks statistical background to support it.

**FIGURE 3:**
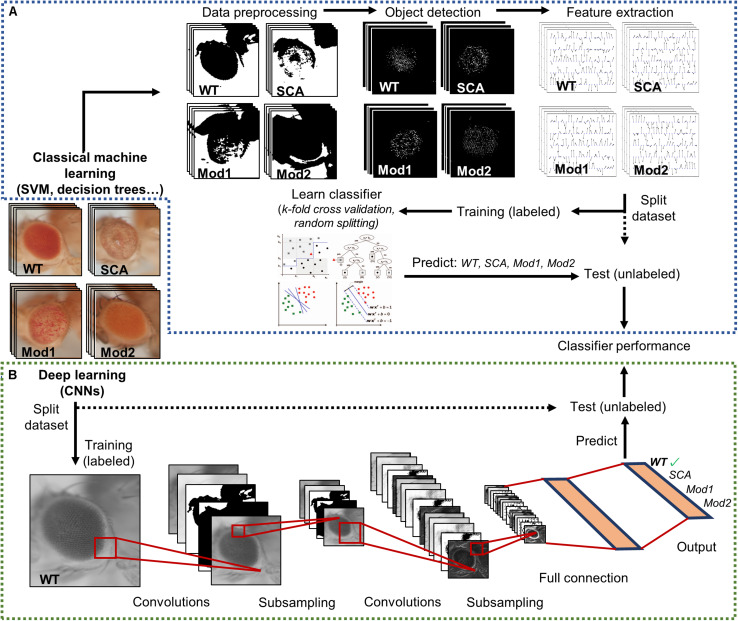
Supervised image classification pipelines. Both workflows start with a dataset labeled with predefined classes. A final performance assessment is also mandatory to test whether the classifier is able to generalize to independent datasets. **(A)** Conventional machine learning methods heavily depend on raw data preprocessing. Splitting into training and test sets occurs only after relevant features have been extracted from the curated data. **(B)** Deep learning techniques receive raw pixel intensities directly as input, so the pipeline begins by splitting the datasets. A simple CNN architecture is depicted as an example. Relevant feature representation occurs in the inner layers of the network, after subsequent convolution and pooling steps. Adapted from Tarca et al. (2007) and Sommer and Gerlich (2013).

Hence, there is a need to tackle a fully automatized, statistically multivariate assessment of *Drosophila* eye’s quantification, given its utmost relevance as a simple, yet comprehensive, model for testing general biology hypotheses and human neurological diseases. Particularly, machine learning algorithms have proven to be incredibly efficient image classifiers during the past decade (Bishop, 2006), rapidly permeating in the fields of cell biology and biomedical image-based screening (Sommer and Gerlich, 2013; Chessel, 2017; Tyagi, 2019). Machine learning methods greatly ease the analysis of complex multi-dimensional data by learning processing rules from examples that can be later on generalized to classify new, unseen data (Figure 3A).

The machine learning techniques typically applied to image classification includes support vector machines (SVM) (Ben-Hur et al., 2008; Chauhan et al., 2019), decision trees (DT) (Orrite et al., 2009), random forests (RF) (Schroff et al., 2008), and neural networks (NNs) (Giacinto and Roli, 2001). Alongside processing power and graphic card-dedicated coding, deep learning methods have exponentially grown in importance during the last few years (LeCun et al., 2015; Po-Hsien et al., 2015). The conventional machine learning algorithms aforementioned require data processing and feature enrichment prior to the training phase as they are not suited to work with raw input. In contrast, the deep learning procedures are general-purpose learners in the sense that they can be fed with raw data, automatically suppress irrelevant information, and select discriminant characteristics, composing simple layers of non-linear transformations into a higher, more abstract level of representation (Figure 3B). The convolutional neural networks (CNNs) are a well-known architecture for deep learning and have been continuously outperforming the previous machine learning techniques, especially in computer vision and audio recognition (Po-Hsien et al., 2015). With the increasing availability of large biological datasets, its popularity in bioinformatics and bioimaging has quickly escalated, and currently CNNs are addressing problems hardly resolvable by former top-notch analysis techniques (Angermueller et al., 2016; Chen et al., 2016; Kraus et al., 2016; Spanhol et al., 2016; Yang et al., 2016; Anwar et al., 2018; Badar et al., 2020). The striking advantage of these networks is that a feature’s hand-crafting and engineering are completely avoided as they implement functions insensitive to perturbations, thanks to the multilayer mapping representation of discriminant details.

The novelty of the present work consists in applying and comparing the different image classification strategies mentioned so far in an extensively used biological model, *D. melanogaster*, which has been scarcely addressed before and is in dire need of a state-of-the-art quantification framework.

## Materials and Methods

### Fly Lines and Maintenance

All stocks and crosses were grown in a temperature-controlled incubator at 25°C, 60% relative humidity, and under a 12-h light–dark cycle. They were fed on conventional medium containing wet yeast 84 g/L, NaCl 3.3 g/L, agar 10 g/L, wheat flour 42 g/L, apple juice 167 ml/L, and propionic acid 5 ml/L. To drive transgene expression to the eye photoreceptor, we used the line gmr:GAL4. Rough eye phenotype was triggered using the UAS:hATXN182Q transgene (Fernandez-Funez et al., 2000) that models human type 1 spinocerebellar ataxia (SCA1), and different UAS:modifier-gene constructs were used to test the system capability to recognize intermediate phenotypes.

### Sample Size

A total of 308 image files were saved using NIS-Elements software in TIFF format. The number of pictures by category is as follows: 82 wild type (WT), 44 gmr > SCA1, 55 modifier #1, 62 modifier #2, and 65 modifier #3.

### External Eye Surface Digital Imaging

Digital pictures (2,880 × 2,048 pixels) of the surface of fly eyes were taken with a Nikon DS-Fi3 digital camera and viewed with a Nikon SMZ1000 stereomicroscope equipped with a Plan Apo× 1 WD70 objective. The flies were anesthetized with CO_2_ and their bodies were immobilized on dual adhesive tape, with their heads oriented to have an eye parallel to the microscope objective. The fly eyes were illuminated with a homogeneous fiber optic light passing through a translucid cylinder so that the light rays were dispersed and did not directly reach the eyes. The images taken with this method show a better representation of the surface retinal texture in contrast to the pictures where light fell upon the eye and the lens’ reflection was captured by the camera, forming bright-spotted grids. The additional settings include an 8× optical zoom in the stereomicroscope.

### ROI Selection Algorithm

All image analyses were performed using R programming language (R Core Team, 2018). The eye images in red/green/blue (RGB) color space were first resized to one-fourth of their original resolution to help fit the image data to the memory capacity of the computer system used. White TopHat morphological transformation with a disc kernel of size 9 was applied using the package EBImage (Pau et al., 2010). The transformed images are converted to grayscale and thresholded to keep only pixels with intensity >0.99 quantile. The overall centroid of the remaining pixels is estimated using the Weiszfeld L1-median (Vardi and Cun-Hui, 2000). For each pixel, the Euclidean distance to the centroid is calculated, and those with distances >0.8 quantile are discarded. A 0.90 confidence level ellipse is estimated on the final selected pixels, and its area is superimposed to the original resized picture to extract the final ROI.

### HOG Descriptor and Machine Learning Classifiers

Firstly, RGB ROIs were converted to grayscale while maintaining the original luminance intensities. The histogram of gradient (HOG) features was extracted using the OpenImageR package (Mouselimis, 2017). A 5 × 5 cell descriptor with five orientations covering a gradient range of 0–180° was estimated per cell in the gradient, resulting in a final 125-dimensional vector for each ROI.

The SVM, DT, and RF algorithms were trained on the extracted HOG features. The dataset was split into training and test sets with a 75/25 ratio using stratified random sampling to ensure class representation. The modeling strategy for all classifiers included cross-validation to assess generalization, grid search for parameter selection and performance evaluation on test set *via* confusion matrix, global accuracy, Kappa statistic, and multiclass pairwise area under the curve (AUC) (Ferri et al., 2003). We tested a radial basis function (RBF) kernel SVM, DT, adaptative boosting DT, and 1,000-trees RF using the R packages *kernlab*, *C50*, and *caret* (Karatzoglou et al., 2004; Kuhn et al., 2015).

### Deep Learning Classifiers

The extracted ROIs were resized to a 224 × 224 × 3 RGB array and stored in vectorized form, resulting in a final data frame of 308 × 150,528 dimensions. The dataset was split into training and test sets with a 75/25 ratio using stratified random sampling to ensure class representation. We further confirmed that the training and test partitions were representative of the sample variability *via* a loss plot (Supplementary Figure S1). Two CNNs were trained on this data:

(i) A simple CNN trained from scratch, with hyperbolic tangent as activation function, two convolutional layers, two pooling layers, two fully connected layers (200 and five nodes), 30 epochs, and a typical softmax output. Each convolutional layer uses a 5 × 5 kernel and 20 or 50 filters, respectively. The pooling layers apply a classical “max pooling” approach. All the parameters in kernels, bias terms, and weight vectors are automatically learned by back-propagation with a learning rate equal to 0.05 and a stochastic gradient descent (SGD) optimizer to ensure that the magnitude of the updates stayed small (Bottou, 2010).
(ii) A fine-tuned CNN using an ImageNet pretrained model with a batch-normalization network structure (Deng et al., 2009; Ioffe and Szegedy, 2015), 30 epochs, a very slow learning rate (0.05), and a SGD optimizer. The final fully connected (five nodes) and softmax output layers are tuned to fit the new fly eye ROIs.

For the CNN training, the R package *MXNet* compiled for the central processing unit (CPU) was used (Tianqi et al., 2015). Performance was assessed in terms of confusion matrix and global accuracy using the *caret* package (Kuhn, 2008).

## Results

### Automatized Detection of *Drosophila* Eye ROIs From Bright-Field Images

The first step in the quantification workflow is the extraction of pixels corresponding to the fly eye from the rest of the image. One concern is that the eye is not flat but convex in morphology, so under white light only the central surface is at the camera focus. To address this issue, white TopHat morphological transformations were performed, defined as the difference between the input image and its opening by a structuring kernel. The opening operation involves erosion followed by a dilation of the image, retrieving the objects of the input image that are simultaneously smaller than the structuring element and brighter than their neighbors.

**FIGURE 4:**
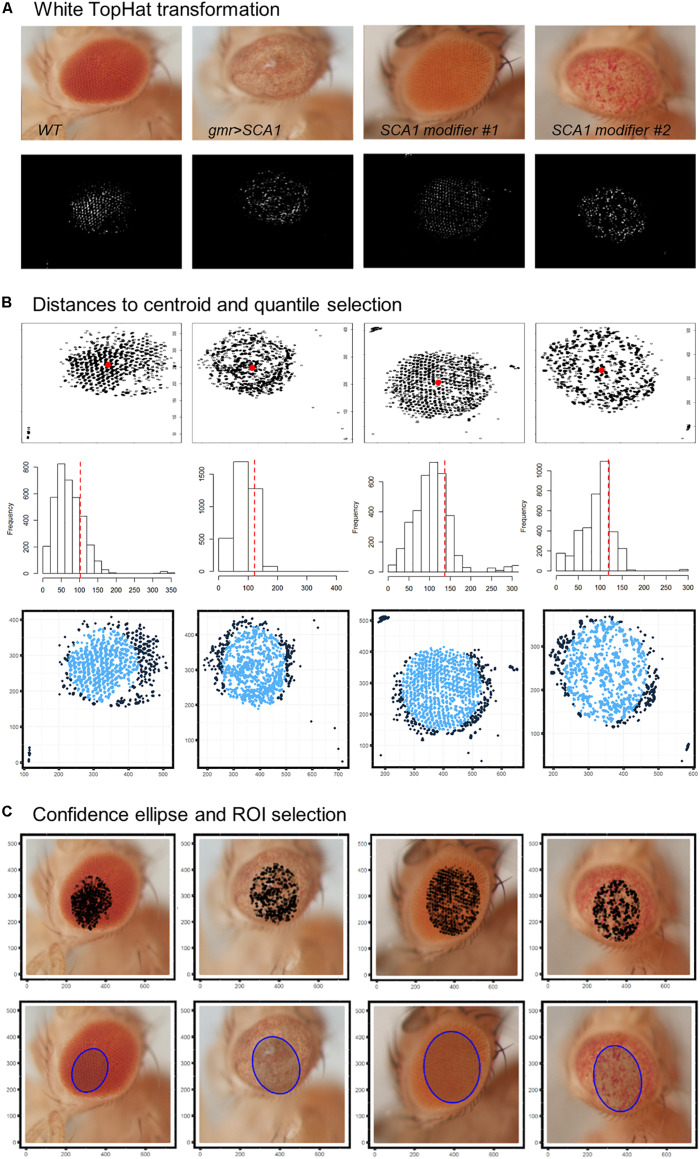
*Drosophila* eye ROI detection strategy. Representative examples of healthy and degenerated eyes are shown. **(A)** Morphological transformation and intensity thresholding extract pixels mostly contained within the eye. **(B)** Euclidean distance to the centroid (red dot) and frequency histogram for quantile selection. Dark blue points are discarded as potential pixel outliers outside the eye limit. **(C)** Selected pixels are superposed to the original image and those within the area of a 0.90 confidence ellipse are extracted as the final ROI (blue shaded ellipse).

**FIGURE 5:**
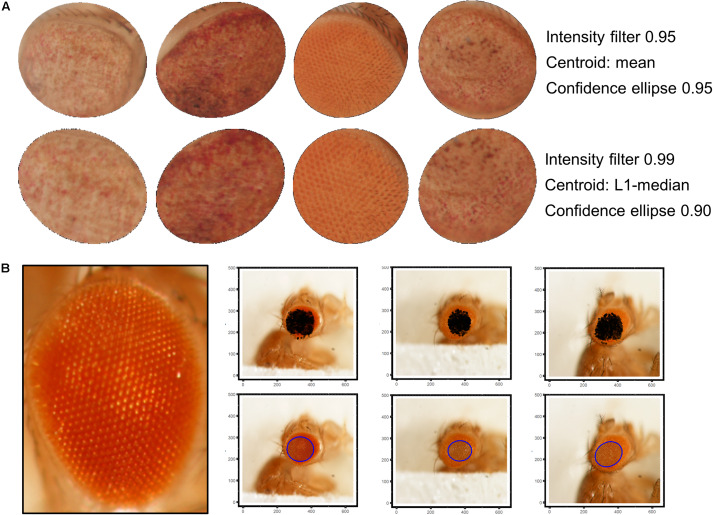
ROI selection optimization and extensibility. **(A)** L1-median centroid alongside stricter thresholds improve the eye area detection. **(B)** Bright-spotted fly eye images can also be successfully segmented using this method.

Best results were obtained using a 9 × 9 disc-shaped kernel followed by a thresholding of pixels with intensities over the 0.99 percentile (Figure 4A). Afterward, the centroid of the selected pixels was calculated as the L1-median, which is a more robust estimator of the central coordinates than the arithmetic mean. Points with Euclidean distance to the centroid greater than 0.8 percentile are more likely to lie outside the eye area and were discarded (Figure 4B). A 0.90 confidence ellipse calculated on the selected pixels conforms the area of the final ROI, which was superimposed and cropped from the original eye image (Figure 4C). As can be appreciated in the example images, the method is invariant to the location of the eye within the image. Various combinations of the thresholds and the centroid estimator were tested (Figure 5A). The proposed segmentation method also works well on bright-field images where light falls directly onto the ommatidium and the eye is seen as a region enriched in reflection spots (Figure 5B). The full array of the final ROIs is represented in Supplementary Figure S2.

We also addressed whether there was any anatomical preference in the ROI extraction that could be biasing the classification procedure. To this extent, heatmaps overlaying all the elliptic ROIs were generated genotype-wise (Supplementary Figure S3). There were a few instances in which the selected ROI included areas outside the eye. This happened more easily in the modifier genotypes and can be seen in the figure as shades that lay outside the eye border. Overall, the ROI extraction seems to be robust against the different ommatidial distribution and eye shapes.

### HOG Feature Extraction

Region of interests cannot be directly input to classical machine learning techniques, so the information contained in their pixels must be extracted beforehand. This is done by estimating a HOG, which can be interpreted as a feature descriptor of a picture that outputs summarized information about predominant shapes and structures. The HOG technique starts by dividing the picture into cells and identifying whether a given cell is an edge or not. HOG provides the edge direction as well, which is done by extracting the gradient and orientation (magnitude and direction) of the edges across neighbor cells. These cells comprise the local regions of related pixels, from which the HOG generates a histogram using the gradients and the orientations of the pixel values, hence the name “histogram of oriented gradients.”

Prior to the HOG extraction, the ROIs were transformed to grayscale, preserving the luminance of the original RGB image. Then, a 125-dimensional feature vector is extracted for each ROI, representing the frequency of a certain gradient within the image (Supplementary Figure S4). The matrix formed by the 125-D vectors of all ROIs conforms to the input for the machine learning classifiers.

Note that HOG is only used to feed the classical machine learning algorithms (SVM, DT, and RF), not the deep learning CNN, which directly uses the pixels’ values as input. This is due to the internal structure of the CNN, the inner layers of which serve as border and edge detectors themselves. This is a reason that led us to believe that pigmentation was not affecting the classification procedure, given that all the methods we used relied on structure detectors rather than color differences.

### Comparison of Machine Learning Classifiers

**FIGURE 6:**
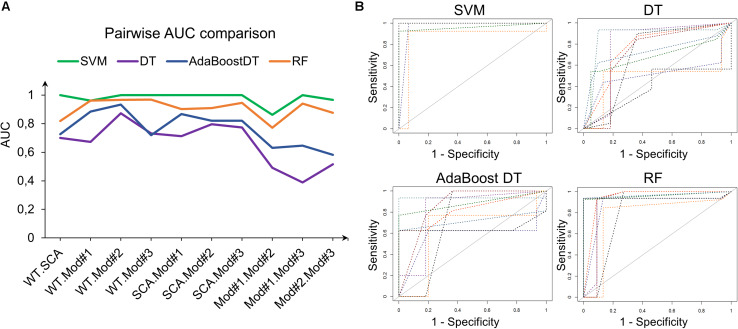
Class pairwise AUC and ROC. **(A)** SVM with RBF kernel outperforms the other classifiers in all comparisons. **(B)** ROC plots corresponding to the AUCs in **(A)**.

Support vector machine with RBF kernel, DT, AdaBoost DT, and 1,000-trees RF algorithms were tested on the extracted HOG features. The sample consisted in 308 fly eye images distributed in five different phenotype classes with varying degrees of retinal surface degeneration. The data were split using stratified random sampling in 75% training and 25% test set. The optimal parameters for each classifier were found using 10-fold cross-validation on the training set. Table 1 shows the confusion matrix, and Table 2 represents the global accuracy, Kappa statistic, and multiclass AUC, defined as the average AUC of class pairwise comparisons (Figure 6A), which was calculated on the test data. The pairwise receiver operating characteristic (ROC) plots are represented in Figure 6B.

**TABLE 1:** Machine learning classifier confusion matrix.

	**Reference**																			
**Predicted**	**WT**				**gmr > SCA**				**SCA modifier #1**				**SCA modifier #2**				**SCA modifier #3**			
WT	20	16	20	20	0	2	2	2	1	4	3	1	0	0	0	1	0	4	3	1
gmr > SCA	0	2	0	0	11	7	7	8	0	2	0	0	0	1	1	0	0	2	3	0
SCA modifier #1	0	0	0	0	0	0	2	0	12	7	10	11	0	0	0	0	0	0	0	0
SCA modifier #2	0	1	0	0	0	0	0	0	0	0	0	1	14	12	11	12	0	3	0	0
SCA modifier #3	0	1	0	0	0	2	0	1	0	0	0	0	1	2	3	2	16	7	10	15

*Colors scheme: SVM, DT, Boost DT, RF.*

**TABLE 2:** Machine learning performance evaluation metrics on test data.

	**Classifier**			
**Metric**	**SVM RBF**	**DT**	**AdaBoost DT**	**1000 RF**
Accuracy	0.973 (0.907–0.997)	0.653 (0.535–0.760)	0.773 (0.662–0.862)	0.880 (0.784–0.944)
Kappa	0.966	0.560	0.711	0.847
Multiclass AUC	0.978	0.665	0.763	0.906

*True positives are shaded in gray.*

In general, the four classifiers performed fairly well on unseen data. Both DT algorithms fell on the low spectrum either in accuracy and AUC (<0.80), whereas RF achieved a remarkable AUC of 0.90. Overall, SVM accomplished the best results among all the error metrics evaluated, with a global accuracy of 0.97 (0.90–0.99), Kappa of 0.96, and a multiclass AUC of roughly 0.98. The parameters that yield these results were a Gaussian kernel, a cost penalty = 1, and sigma = 0.005. The WT eyes were the most correctly classified phenotype by the four methods.

**FIGURE 7:**
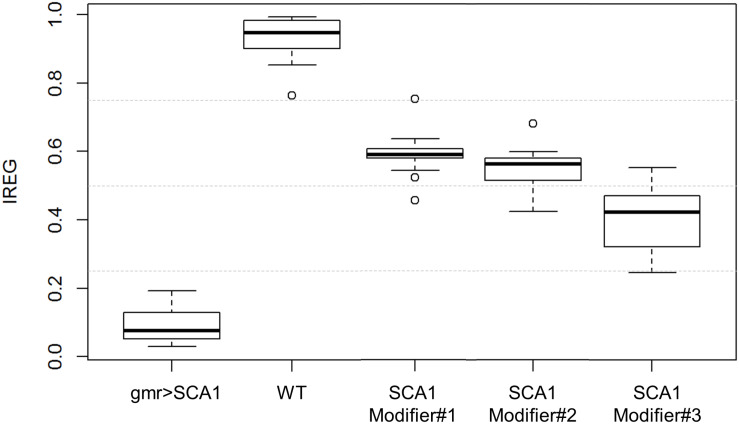
IREG boxplots. WT and SCA1 eyes show opposing IREG values and no distribution overlap. SCA modifiers show intermediate rough eye phenotypes and slight distribution overlapping, but the median and central boxes differentiate them. Gray dotted lines mark 0.25, 0.5 and 0.75 IREG values. Sample sizes are as follows: 82 WT, 44 gmr > SCA1, 55 modifier #1, 62 modifier #2, and 65 modifier #3.

From the SVM estimated class probabilities, it is possible to derive a IREG that ranges from 0 (total degeneration) to 1 (healthy eye) (Diez-Hermano et al., 2015). It is based on the knowledge of the degeneration intensity of the phenotypes involved in the model: WT < modifier #1 < modifier #2 < modifier #3 < SCA1, from absence to full presence of rough eye phenotype. IREG is then calculated as:

$$\text{IREG}=\frac{\overset{4\cdot P\left(\text{eye}=\text{WT}\right)+3\cdot P\left(\text{eye}=\text{Mod}\#1\right)}{+2\cdot P\left(\text{eye}=\text{Mod}\#2\right)+P\left(\text{eye}=\text{Mod}\#3\right)}}{4}$$

when estimated on the test data, the IREG distribution fits to the expected values and properly reflects the intrinsic variability of the fly model and the rough eye phenotype (Figure 7).

### Deep Learning Classifiers

In contrast with the previous machine learning classifiers that needed a transformation of the cropped images into an enriched feature space (HOG), deep learning algorithms directly use the ROI pixel intensity arrays as input. The features are automatically learned during the learning process, from gross edge and contour detection to discrimination of fine details the deeper the layer in the network.

**FIGURE 8:**
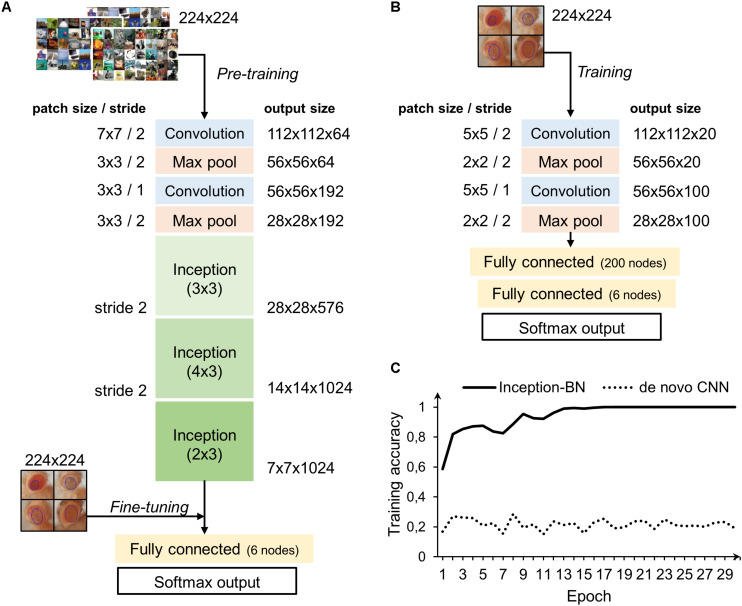
CNN architectures and learning curve. **(A)** Inception-BN is a 15-layered CNN pre-trained on thousands of natural images. A six-nodes fully connected layer and softmax output are trained with new fly eye images on top of the Inception blocks. **(B)** *De novo* CNN with five layers and a six-nodes fully connected and softmax output. **(C)** Training accuracy in the pre-trained model starts pretty high and quickly rises in the first few epochs. In contrast, *de novo* model accuracy remains low and fluctuates around the initial value with no apparent signs of improvement.

Two different strategies were followed to train the deep networks: learning a *de novo* model and transfer learning. The latter approach takes advantage of CNNs pre-trained on very large samples, which is especially well suited for classifying new small datasets as the majority of patterns and motifs commonly found in the images are already known to the model internal representation. Thus, it is only necessary to fine-tune the final layers to learn the particularities of the new images, which is many times faster than training a CNN from scratch and does not require thousands of labeled examples. The architectures of both *de novo* and pre-trained CNN are depicted in Figures 8A,B. The pre-trained model chosen uses the inception structure, characterized by including mini-batch normalization (BN) for each training epoch, which allows for high learning rates and acts as regularizer. In comparison, the *de novo* CNN is much shallower due to computational constraints.

Accuracy during the training phase is usually a reliable indicator of a CNN capability to learn the discriminative features with the available sample size (Figure 8C). The curve of the *de novo* CNN is a clear sign that either the network is not deep enough or the training sample is too small for the complexity of the classification task at hand. One major concern with the pre-trained inception-BN was the possibility that the network was memorizing the training set, given the few epochs it needed to achieve perfect training accuracy. A performance assessment in an independent test set of unseen images gave impressive accuracy and AUC values close to 1 (Tables 3, 4), refuting the possibility of overfitting. The CNN trained from scratch predicted every eye to be WT, indicative of the weak classifying rule learned in training.

**TABLE 3:** Convolutional neural networks classifier confusion matrix.

	**Reference**									
**Predicted**	**WT**		**gmr > SCA**		**SCA modifier #1**		**SCA modifier #2**		**SCA modifier #3**	
WT	20	20	0	11	0	13	0	15	0	16
gmr > SCA	0	0	11	0	0	0	0	0	0	0
SCA modifier #1	0	0	0	0	13	0	0	0	0	0
SCA modifier #2	0	0	0	0	0	0	14	0	0	0
SCA modifier #3	0	0	0	0	0	0	1	0	16	0

*Color scheme: Inception-BN *de novo* CNN.*

**TABLE 4:** Convolutional neural networks performance evaluation metrics on test data.

	**Classifier**	
**Metric**	**Inception-BN**	***De novo*** **CNN**
Accuracy	0.986	0.146
Multiclass AUC	0.997	0.5

*True positives are shaded in gray.*

Transfer learning CNN was also the only model capable of properly quantifying the pictures taken under drastically different illumination techniques, relative to the sample that it was trained on (dispersed indirect light). The genetic background also differed from the training sample and corresponds to tau-related neurodegeneration, the accumulation of which contributes to the pathology of Alzheimer disease (Lasagna-Reeves et al., 2016; Rousseaux et al., 2016; Galasso et al., 2017). Supplementary Figure S5 shows how IREG estimations coming from CNN were more representative of the surface regularity than IREG coming from the SVM, which was the top-performance machine learning technique. In fact, the SVM predicted all eyes to have essentially the same IREG value. Additionally, retraining all models with our original sample of bright-spotted eyes (Supplementary Figure S2B) resulted in transfer learning CNN being the only model that could successfully generalize to a set of new eyes (Supplementary Table S1).

Given its classification accuracy and versatility, transfer learning with the pre-trained inception-BN model is arguably the top performer classifier among all the methods tested in this work.

## Discussion

The present work provides a novel and fully automated method to quantitatively assess the degeneration intensity of the fruit flies’ compound eye using reliable and robust state-of-the-art machine learning techniques. This new method consists in the acquisition of bright-field images from the external retinal surface, the automatic extraction of a ROI enriched in information of the eye morphology, and a classification algorithm built around a pre-trained deep learning algorithm, fine-tuned to the particularities of the eye degeneration’s images. Additionally, a model based on the combination of HOG features extraction and Gaussian kernel SVM offered performance on par with the CNN and, in fact, required much less training time.

In contrast with previous quantification approaches (Caudron et al., 2013; Diez-Hermano et al., 2015; Iyer et al., 2016), this method does not rely on patterns that are created by light reflecting in the eye lenses, so it can be applied to extract ROIs from a variety of illumination conditions. While performing the experiment to validate the method, we have estimated the total time that it takes for a researcher to analyze an experimental group of 50 flies: 1.5 h from anesthetizing the flies until the final IREG plot was statistically assessed. It is noteworthy that the most part of that estimation was devoted to capturing the images, which is a mandatory step, whether the flies are to be manually or automatically classified later on. The proposed pipeline can process a 2,880 × 2,048-resolution image in less than 10 s and batches of 50 images in approximately 90 s, depending on the hardware that it runs on.

One of the major goals of this work was to analyze the potentiality of deep learning techniques to extract feature maps directly from the raw pixel array, which could be used as input to other conventional machine learning algorithms (i.e., SVM). Due to computational constraints, it was not possible to tune up the graphics processing unit-compiled versions of the software utilized, and the prohibitive CPU computational time and memory usage in its absence made the evaluation of the former objective not feasible. HOG was chosen as an alternative descriptor, given its successful application in object detection (Dalal and Triggs, 2005; Orrite et al., 2009; Li et al., 2012), and ended up resulting in a surprisingly powerful classifier in combination with conventional SVM. Nonetheless, the pre-trained CNN will still be preferable for pictures taken under illumination conditions different from the ones the models were trained on as it has been shown to have greater discriminative power.

A drawback of CNNs is the staggering amount of labeled training examples that they need to learn adequate internal representations of image patterns and motifs. Although the sample size in *Drosophila* experiments ranks among the largest of any animal model in genetics, it is still a titanic effort to go beyond 1,000 images in a typical fruit fly assay. This limitation affected the performance of the *de novo* CNN, which led to the alternative strategy of transfer learning. Using inception-BN, a CNN pre-trained on millions of natural images (Ioffe and Szegedy, 2015), proved to be a well-thought solution that definitely opens up the field of deep learning to small-scale biology setups.

Future lines of work include developing the fly eye detection algorithm further to make it extensible to other image capturing techniques (i.e., SEM). A more immediate priority is the creation of a user-friendly Shiny application (Winston et al., 2017) that will allow the researcher to tweak the ROI selection parameters to fit the peculiarities of its own dataset prior to the degeneration quantification. Depending on the particular hardware settings, the app may also offer the user the possibility to train its own SVM or deep learning model.

The aim of this work is to provide a workflow that results in a quantitative assessment of the degree of eye degeneration of hundreds of flies in a quick and unbiased manner. This makes our method particularly suitable for discriminating potential genetic rescues or aberrations. We believe that our algorithm could be easily implemented in fully robotized environments as the final quantification step. The highlighted strengths of the proposed framework will enhance the sensitivity of high-throughput genetic screens based on rough eye phenotypes and demonstrate that fly eye imaging is a top-notch technique for the quantitative modeling of human diseases.

## Data Availability Statement

The datasets generated and analyzed for this study, alongside the R scripts, can be found in the FigShare repository at https://tinyurl.com/un8tacu.

## Author Contributions

SD-H performed the experiments, took the fly eye pictures, analyzed the data, and wrote the manuscript. MG and DS designed the genetic background of the flies, took the fly eye pictures, and helped with the manuscript writing. EV-L analyzed the data and helped with the manuscript writing.

## Conflict of Interest

The authors declare that the research was conducted in the absence of any commercial or financial relationships that could be construed as a potential conflict of interest.

## Abbreviations

AdaBoost: adaptative boosting
AUC: area under the ROC curve
BN: batch normalization
CNN: convolutional neural network
DT: decision tree
gmr: glass multimer reporter
HOG: histogram of oriented gradients
IREG: regularity index
MLP: multilayer perceptron
NN: neural network
PolyQ: polyglutaminated
RBF: radial basis function
RGB: red, green, blue (colorspace)
RF: random forest
ROC: receiver operating characteristic
ROI: region of interest
SCA: spinocerebellar ataxia
SEM: scanning electron micrograph
SGD: stochastic gradient descent
SVM: support vector machine
UAS: upstream activating sequence
WT: wild type.
