# Supplementary material for: Machine Learning Representation of Loss of Eye Regularity in a Drosophila Neurodegenerative Model
Source: Front Neurosci. 2020 Jun 4;14:516. doi: 10.3389/fnins.2020.00516 (PMC7287026; doi:10.3389/fnins.2020.00516)
Supplement: Supplementary file 1 [file Table_1.pdf]

Table S1. Models' performance after training with bright-spotted pictures. Evaluation metric on test data. Best results are shaded in grey.

Classifier / Metric	SVM RBF	DT	AdaBoostDT	1000 RF	Inception-BN
**Accuracy**	0.65 (0.531-0.772)	0.34 (0.312-0.503)	0.41 (0.351-0.462)	0.53 (0.412-0.944)	0.85 (0.75-0.90)
**Multiclass AUC**	0.62	0.32	0.432	0.521	0.881
