# Supplementary figures and images for: Machine Learning Representation of Loss of Eye Regularity in a Drosophila Neurodegenerative Model
Source: Front Neurosci. 2020 Jun 4;14:516. doi: 10.3389/fnins.2020.00516 (PMC7287026; doi:10.3389/fnins.2020.00516)

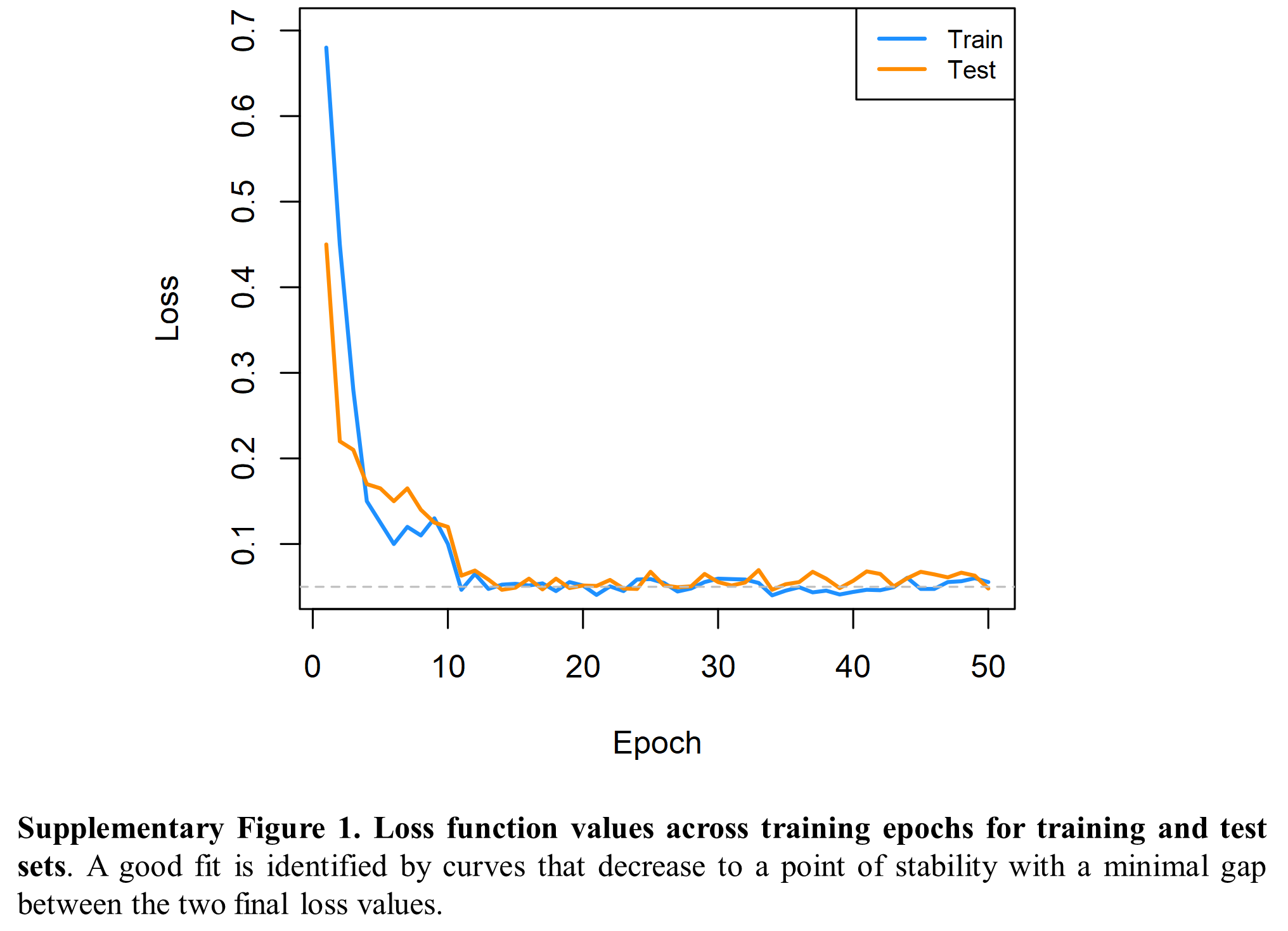

Supplement: Supplementary file 2 [file Image_1.tif]

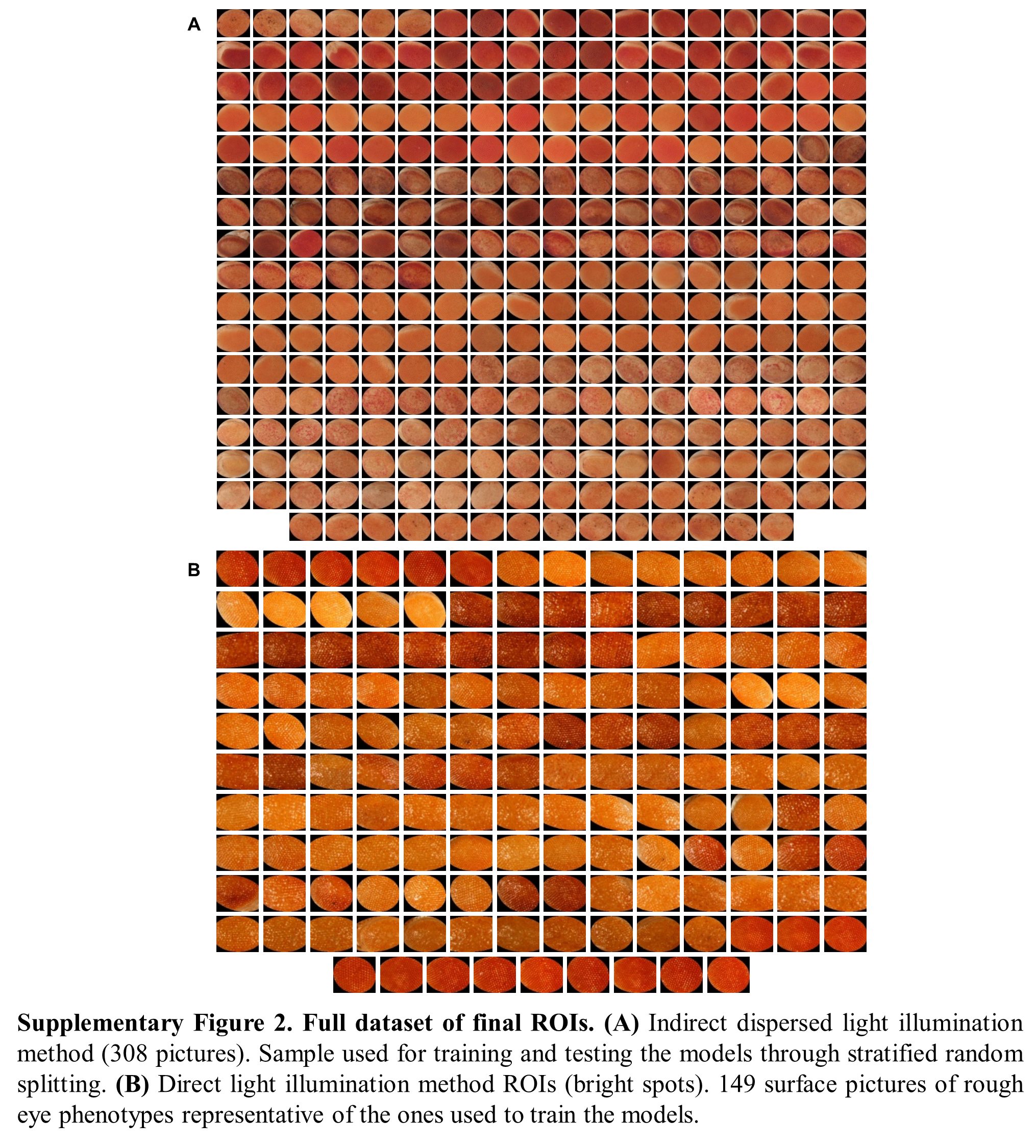

Supplement: Supplementary file 3 [file Image_2.tif]

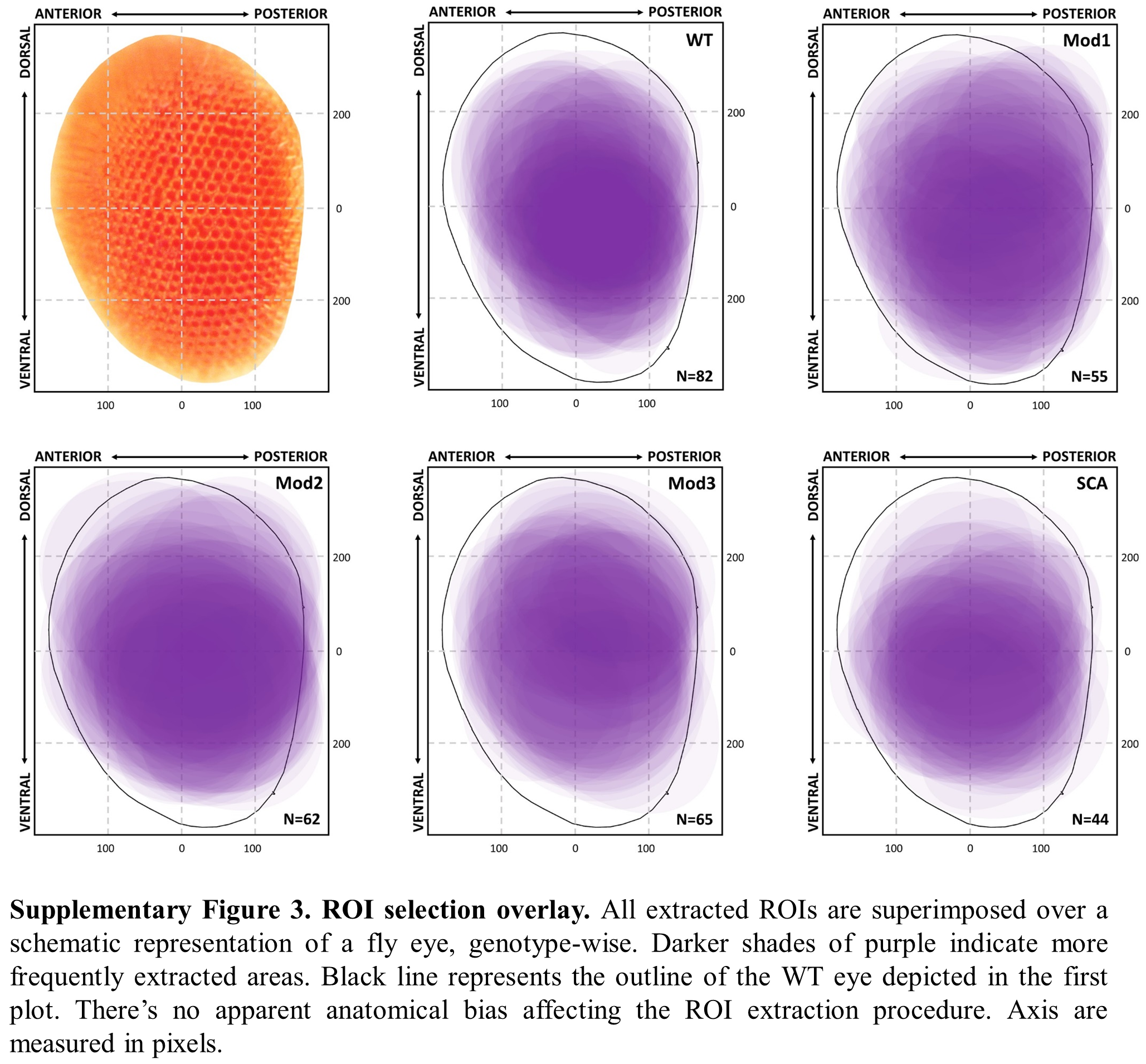

Supplement: Supplementary file 4 [file Image_3.tif]

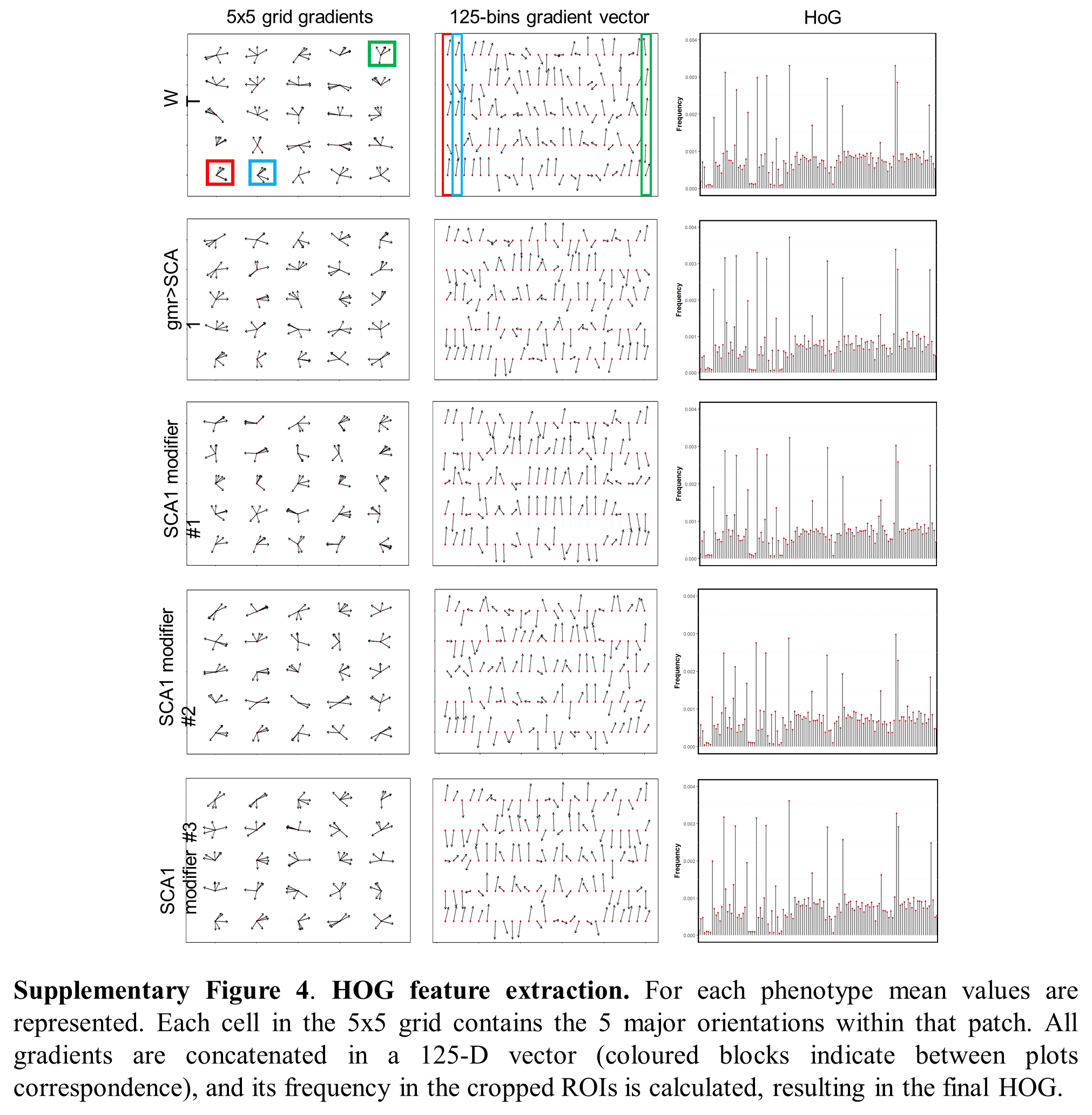

Supplement: Supplementary file 5 [file Image_4.tif]

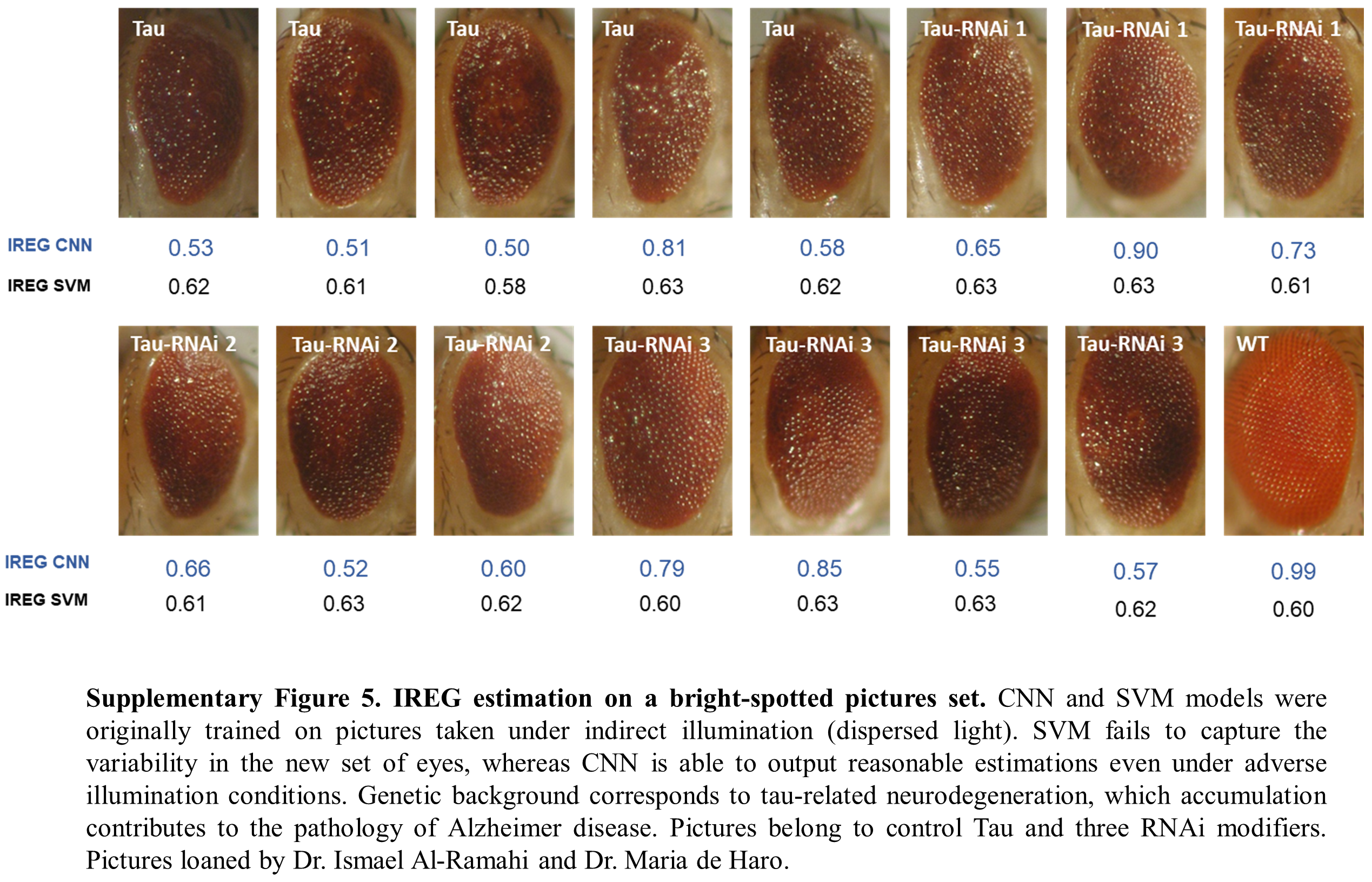

Supplement: Supplementary file 6 [file Image_5.tif]
